# Severe Fournier's Gangrene Complicated by Polymicrobial Bacteremia and Persistent Abdominal Wall Fluid Collections Requiring Readmission

**DOI:** 10.7759/cureus.115275

**Published:** 2026-08-27

**Authors:** Garrett F Sakamoto, Ariann Howard, Rebecca Tsai, Peyman Arghavanifard

**Affiliations:** 1 Department of Internal Medicine, Dignity Health California Hospital Medical Center, Los Angeles, USA; 2 Department of Internal Medicine, Ross University School of Medicine, Bridgetown, BRB

**Keywords:** abdominal-wall fluid collections, bacteremia, case report, diabetes mellitus, fournier's gangrene, necrotizing fasciitis

## Abstract

Fournier's gangrene is a rapidly progressive necrotizing soft tissue infection of the perineal and genital regions associated with significant morbidity and mortality. Patients with poorly controlled diabetes mellitus are at particularly high risk for severe disease and complications. We present the case of a 57-year-old Hispanic male with uncontrolled type 2 diabetes mellitus (hemoglobin A1c of 12.2%), hypertension, and alcohol use disorder who presented with a two-week history of worsening scrotal pain and swelling. Computed tomography demonstrated extensive subcutaneous emphysema involving the perineum, scrotum, penis, bilateral inguinal canals, and anterior abdominal wall. The patient underwent multiple operative debridements, end colostomy creation, and prolonged antimicrobial therapy for polymicrobial bacteremia and severe sepsis. His hospital course was further complicated by bilateral abdominal wall fluid and gas collections concerning for abscess formation, requiring image-guided drainage and subsequent readmission for persistent collections. After a cumulative hospital stay of approximately seven weeks, he was ultimately discharged in stable condition. This case highlights the aggressive nature of Fournier's gangrene and the importance of early surgical intervention, repeated source control, and close follow-up for persistent or delayed infectious complications.

## Introduction

Fournier's gangrene is a rare but potentially fatal necrotizing soft tissue infection involving the perineal, perianal, and genital regions [1]. The disease spreads rapidly along fascial planes, resulting in tissue necrosis, systemic toxicity, and significant morbidity and mortality without early recognition and treatment [1,2].

Diabetes mellitus is the most commonly reported predisposing condition and has been associated with increased disease severity and poorer outcomes [3,4]. Additional risk factors include chronic alcohol use, obesity, malignancy, and other forms of immunosuppression [1,3]. Management requires prompt resuscitation, broad-spectrum antimicrobial therapy, and aggressive surgical debridement, which remains the cornerstone of treatment [2,5].

We present a severe case of Fournier's gangrene in a patient with uncontrolled diabetes mellitus and polymicrobial bacteremia who required multiple operative debridements and end colostomy creation. Although abdominal wall extension may occur in advanced Fournier's gangrene, the development and persistence of bilateral fluid and gas collections following initial surgical source control represent a clinically important complication requiring continued surveillance. In this patient, these collections ultimately required image-guided drainage, readmission, and continued antimicrobial therapy.

## Case presentation

A 57-year-old Hispanic male with poorly controlled type 2 diabetes mellitus (hemoglobin A1c 12.2%), hypertension, and alcohol use disorder presented with a two-week history of worsening scrotal pain and swelling accompanied by fevers, chills, nausea, and vomiting.

**Figure 1:**
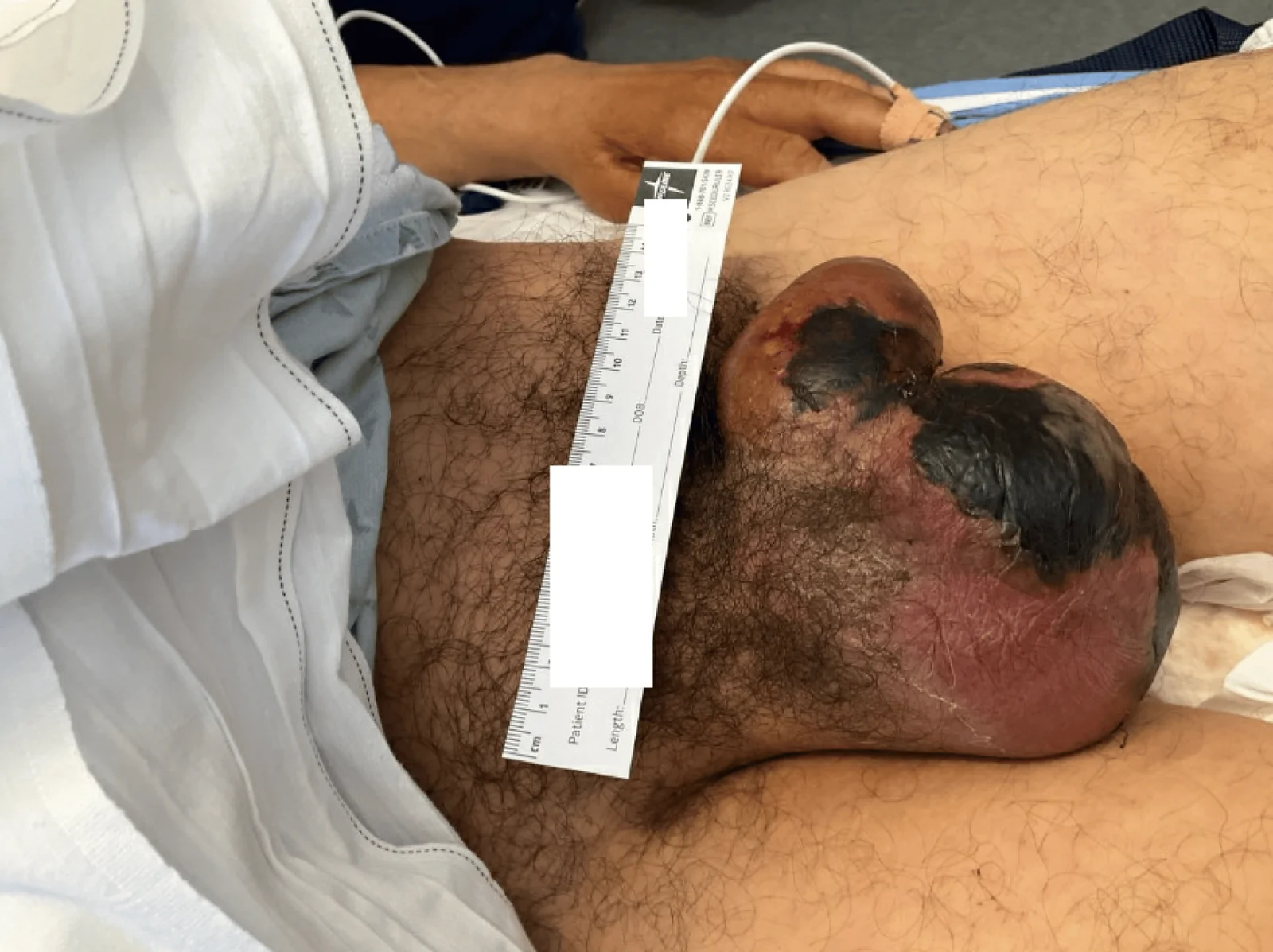
Initial presentation of Fournier's gangrene Clinical photograph obtained at presentation demonstrating extensive necrosis of the scrotum and perineum prior to operative debridement. Written informed consent was obtained from the patient.

On presentation, he was tachycardic to 145 beats per minute. Physical examination revealed foul-smelling necrotic tissue involving the scrotum and perineum with palpable crepitus (Figure 1). Laboratory studies demonstrated marked hyperglycemia, acute kidney injury, metabolic acidosis, elevated inflammatory markers, and lactic acidosis (Table 1). The Laboratory Risk Indicator for Necrotizing Fasciitis (LRINEC) score was 7 [6].

**Table 1:** Laboratory findings on admission LRINEC: Laboratory Risk Indicator for Necrotizing Fasciitis

Laboratory test	Patient value	Reference range
White blood cell count	12.5×10⁹/L	(4.0-11.0)×10⁹/L
C-reactive protein	72.4 mg/dL	<0.5 mg/dL
Lactate	4.7 mmol/L	0.5-2.0 mmol/L
Creatine kinase	413 U/L	30-200 U/L
Glucose	727 mg/dL	70-99 mg/dL
Creatinine	2.7 mg/dL	0.7-1.3 mg/dL
Arterial pH	7.29	7.35-7.45
Bicarbonate	13.9 mmol/L	22-28 mmol/L
Hemoglobin A1c	12.2%	<5.7%
LRINEC score	7	Score ≥6: intermediate-to-high risk

**Figure 2:**
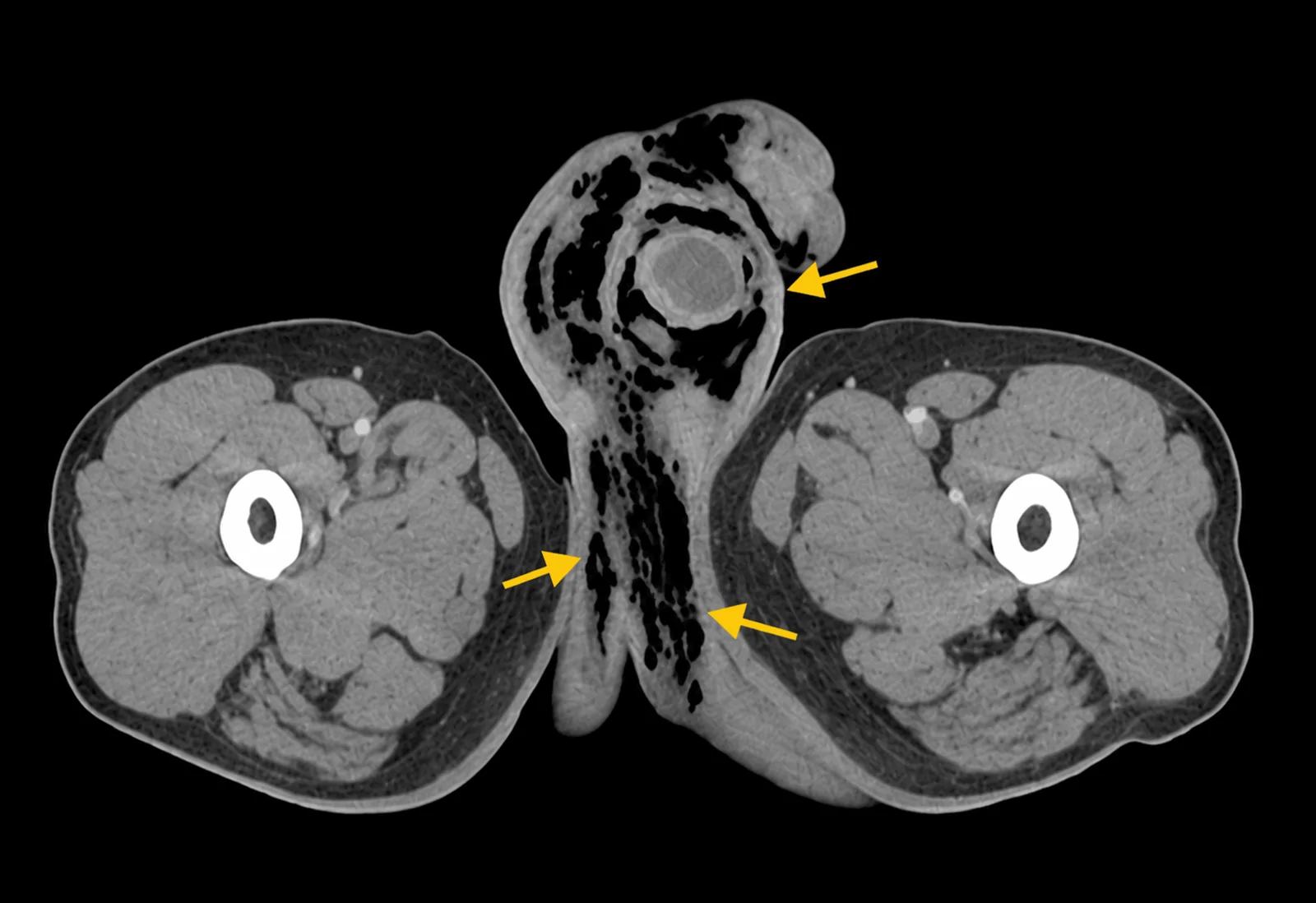
CT findings at presentation Axial contrast-enhanced CT image demonstrating extensive subcutaneous emphysema involving the scrotal and perineal soft tissues with extension along fascial planes (arrows), consistent with necrotizing soft tissue infection in Fournier's gangrene. CT: computed tomography

Computed tomography (CT) of the abdomen and pelvis with intravenous contrast demonstrated extensive subcutaneous emphysema involving the perineum, scrotum, penis, bilateral inguinal canals, and anterior abdominal wall, findings consistent with Fournier's gangrene (Figure 2).

**Figure 3:**
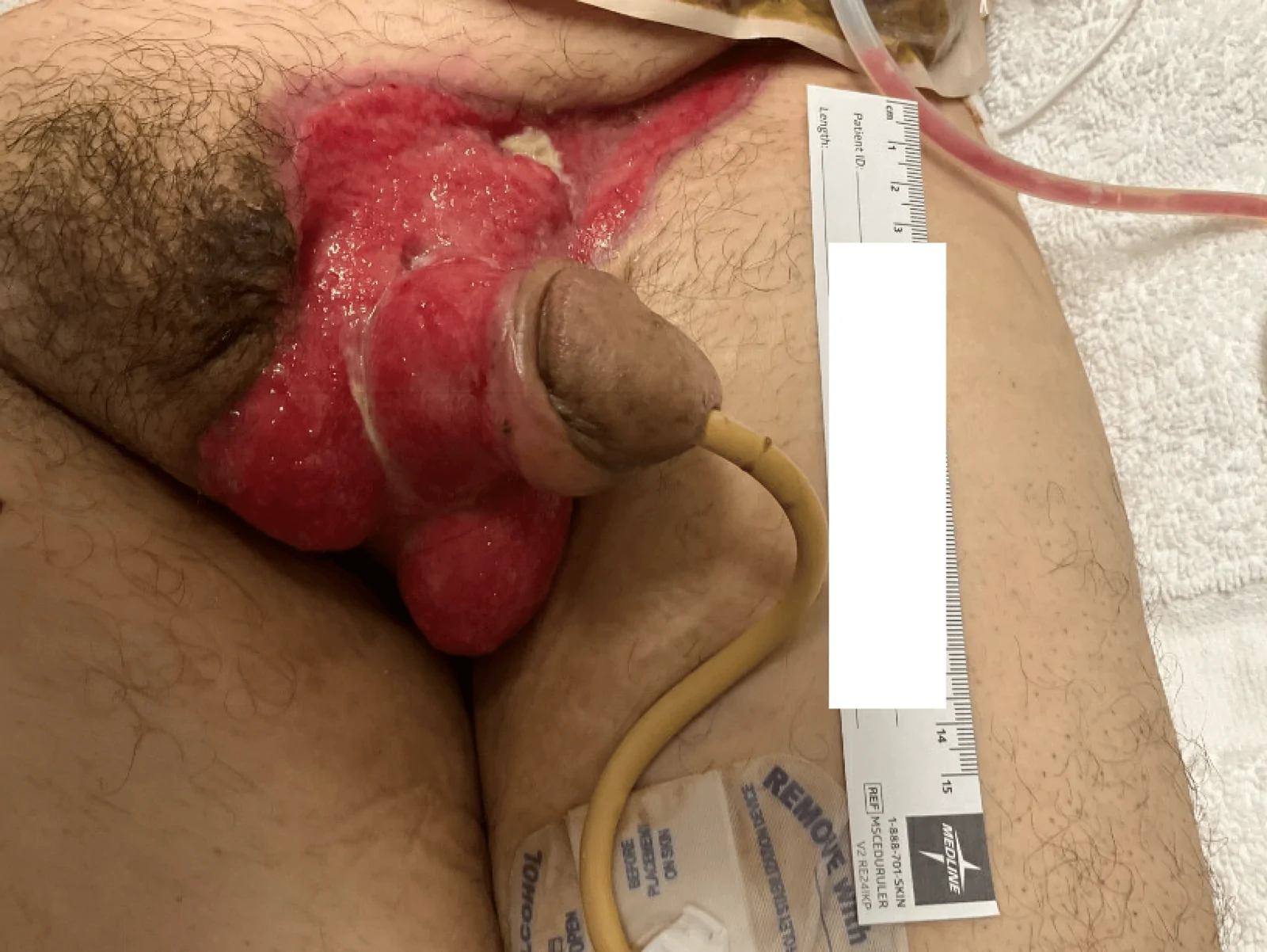
Post-debridement wound following serial operative interventions Clinical photograph obtained following serial operative debridements demonstrating the extent of the resulting soft-tissue defect. Written informed consent was obtained from the patient.

The patient underwent emergent operative debridement on hospital day 1. Wide debridement was performed down to healthy tissue, with both testes exposed and the wound irrigated and packed. On hospital day 2, repeat exploration demonstrated extensive devitalized and necrotic tissue involving the penile shaft, bilateral testes, left inguinal region, anterior abdominal wall, perineum, and inferior buttocks, with purulence extending from the left inguinal region toward the anterior abdominal wall. The involved tissue was sharply debrided to healthy bleeding tissue. Given the extensive perineal involvement and need to limit ongoing fecal contamination of the wound, an exploratory laparotomy with end colostomy creation for fecal diversion was also performed. A third operation on hospital day 7 included further debridement of the sacral wound, perineum, bilateral buttocks, penile shaft, bilateral testes, left inguinal region, and anterior lower abdominal wall. The extent of tissue loss following serial debridements is shown in Figure 3.

The hospital course was complicated by severe sepsis and polymicrobial infection. Initial perineal cultures grew *Klebsiella pneumoniae* and *Bacteroides fragilis*, while blood cultures demonstrated viridans streptococci and *B. fragilis*. The initial *K. pneumoniae* isolate was susceptible to piperacillin-tazobactam and multiple other tested antimicrobial agents. The patient initially received intravenous piperacillin-tazobactam for 14 days and vancomycin for four days; vancomycin was discontinued after methicillin-resistant *Staphylococcus aureus* infection was excluded.

**Figure 4:**
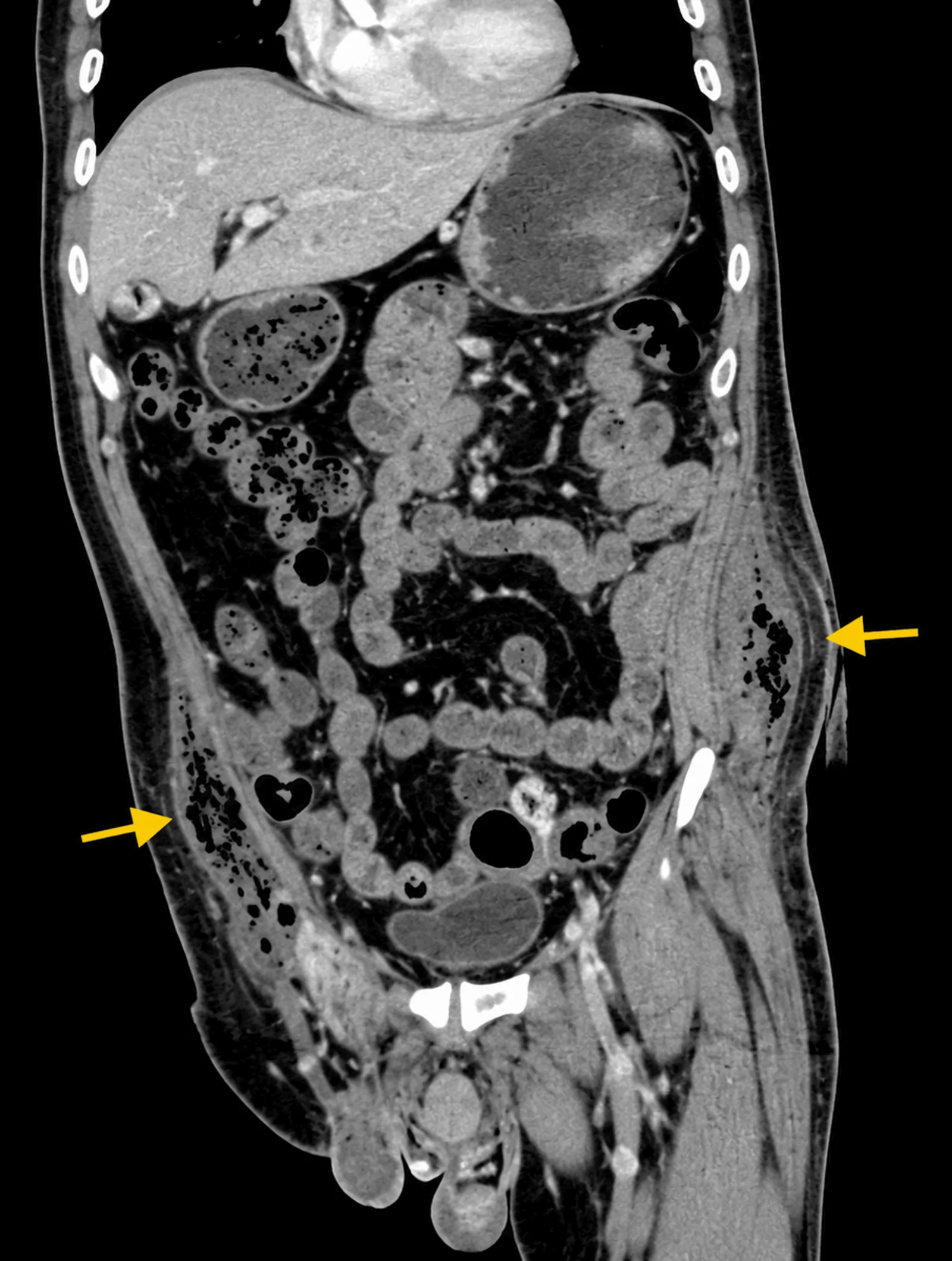
Repeat CT of the abdomen and pelvis Contrast-enhanced coronal CT image demonstrating bilateral lower abdominal wall soft-tissue fluid and gas collections (arrows), concerning for abscesses. Image-guided drainage was subsequently performed. CT: computed tomography

On hospital day 22, repeat CT of the abdomen and pelvis demonstrated lower abdominal wall fluid and gas collections concerning for abscess formation (Figure 4). Ceftriaxone and metronidazole were administered for eight days. Image-guided drainage of the right anterior abdominal collection was subsequently performed with pigtail catheter placement. Culture of the drained fluid grew extended-spectrum beta-lactamase (ESBL)-producing *K. pneumoniae*, which was resistant to piperacillin-tazobactam, ceftriaxone, cefepime, and fluoroquinolones but susceptible to meropenem and ertapenem. Following an episode of fever and worsening leukocytosis, antimicrobial therapy was changed to intravenous meropenem, which was administered for five days. The leukocyte count subsequently decreased from 17.2×10⁹/L to 14.9×10⁹/L. After clinical stabilization and confirmation of susceptibility to ertapenem, therapy was transitioned to ertapenem for three days to facilitate continued antimicrobial therapy at the skilled nursing facility.

After a prolonged hospitalization, the patient was discharged to a skilled nursing facility for continued wound care and rehabilitation. Approximately one month later, he was hospitalized at an outside facility after dislodgement of the right-sided abdominal drain, which was subsequently replaced, and he was subsequently transferred for continued management of persistent abdominal wall and perineal collections. Repeat CT demonstrated substantial improvement in the previously identified collections but persistent scattered rim-enhancing fluid within the perineal and perirectal regions. Interventional radiology placed a right lower-quadrant drain, yielding approximately 3 mL of fluid; Gram stain showed no organisms, and the fluid culture remained negative. A 15-day course of intravenous ertapenem was administered according to infectious disease recommendations, with the final five days completed following discharge. The patient was ultimately discharged in stable condition to a skilled nursing facility with continued intravenous ertapenem, wound care, and follow-up. A summary of the clinical course is provided in Table 2.

**Table 2:** Clinical timeline CT: Computed tomography; ESBL: extended-spectrum beta-lactamase

Hospital day	Clinical event
Day 1	Presentation with Fournier’s gangrene; emergent operative debridement
Day 2	Repeat extensive debridement; exploratory laparotomy and end colostomy creation for fecal diversion
Day 7	Third operative debridement involving the sacral wound, perineum, bilateral buttocks, genital structures, left inguinal region, and anterior lower abdominal wall
Day 22	CT demonstrated bilateral lower abdominal wall fluid and gas collections concerning for abscess formation
Day 25	Image-guided drainage of the right anterior abdominal wall collection with pigtail catheter placement
Day 26	Drainage culture grew ESBL *Klebsiella pneumoniae*
Day 29	Fever and worsening leukocytosis; antimicrobial therapy changed to meropenem
Day 35	Discharged to a skilled nursing facility for continued wound care and rehabilitation
Readmission day 1	Transferred for management of persistent abdominal wall and perineal fluid collections after prior drain dislodgement and replacement
Readmission day 5	Repeat CT demonstrated improvement with persistent rim-enhancing perineal and perirectal fluid collections
Readmission day 9	Right lower-quadrant drain placed by interventional radiology; approximately 3 mL obtained with negative Gram stain and culture
Readmission day 14	Discharged in stable condition with continued ertapenem and wound care

## Discussion

Fournier's gangrene remains a surgical emergency requiring prompt recognition and aggressive intervention [1,2]. Diabetes mellitus is the most commonly reported predisposing condition and has been associated with increased disease severity, prolonged hospitalization, and worse clinical outcomes [3,4]. Our patient presented with severely uncontrolled diabetes mellitus, as demonstrated by an admission hemoglobin A1c level of 12.2%, a well-established risk factor that may have contributed to the extensive disease burden observed at presentation.

Although early surgical debridement remains the cornerstone of treatment, a single operation is often insufficient [2,5]. Despite immediate operative intervention, our patient experienced progressive necrosis involving the perineum, bilateral buttocks, and inguinal canals, ultimately requiring multiple debridements and fecal diversion with end colostomy creation. This clinical course underscores the importance of serial operative reassessment and repeated source control in patients with extensive Fournier's gangrene.

The microbiology of this case was also notable. Perineal wound cultures grew *K. pneumoniae* and beta-lactamase-producing *B. fragilis*, while blood cultures were positive for *B. fragilis* and viridans streptococci. These findings are consistent with the polymicrobial nature of Fournier's gangrene, which commonly involves synergistic aerobic and anaerobic organisms originating from the gastrointestinal and genitourinary tracts [1,7]. Later in the hospitalization, drainage cultures from abdominal wall fluid collections grew ESBL *K. pneumoniae*, prompting escalation to meropenem followed by transition to ertapenem after clinical stabilization.

Although Fournier's gangrene frequently requires serial debridements, this patient's development of bilateral abdominal wall fluid and gas collections, followed by persistent collections requiring readmission and additional image-guided drainage, represented a significant complication of his clinical course. Given the extensive initial spread of infection into the inguinal regions and anterior abdominal wall, the subsequent abdominal wall collections may have represented persistent or evolving infectious foci related to the original necrotizing process. However, a direct causal relationship cannot be definitively established from the clinical course alone. While he initially improved following serial debridements and prolonged antimicrobial therapy, persistent collections required additional drainage and continued treatment. This case highlights the need for continued surveillance even after successful debridement and apparent clinical improvement.

## Conclusions

Fournier's gangrene remains a life-threatening necrotizing soft tissue infection associated with significant morbidity. This case demonstrates the severe complications that may occur in a patient with uncontrolled diabetes mellitus, including polymicrobial bacteremia, multiple operative debridements, fecal diversion, and persistent abdominal wall fluid collections requiring drainage and readmission. Importantly, clinically significant infectious complications may persist or develop despite initial source control and apparent clinical improvement, highlighting the need for continued surveillance and close follow-up.
